# Gender Determination Based on CBCT Maxillary Sinus Analysis: A Systematic Review

**DOI:** 10.3390/diagnostics13233536

**Published:** 2023-11-27

**Authors:** Nikolas Christoloukas, Anastasia Mitsea, Aliki Rontogianni, Christos Angelopoulos

**Affiliations:** 1 Department of Oral Diagnosis and Radiology, School of Dentistry, National and Kapodistrian University of Athens, 11527 Athens, Greece; nchristoloukas@gmail.com (N.C.);; 2 Department of Orthodontics, School of Dentistry, National and Kapodistrian University of Athens, 11527 Athens, Greece

**Keywords:** maxillary sinus, cone-beam computed tomography (CBCT), gender, forensic identification

## Abstract

Gender determination is an essential element for human identification in forensic medicine, to which the maxillary sinuses may contribute as they remain intact even after severe damage to the skull and other structures. Aim: To evaluate scientific evidence published over the last decade to determine whether maxillary sinus dimensions and volume may constitute useful parameters for forensic identification and gender determination, based only on cone-beam computed tomography images (CBCT). Methods: This review adhered to the PRISMA statement’s criteria. Four databases were searched for articles published between January 2010 and April 2023. Results: Initially, 1719 records were identified. After screening, there were 2475 participants in the included studies. Of the fifteen articles selected, five reported data assessing only volumetric measurements of the maxillary sinus, seven reported data calculating only linear measurements and three reported data by combining findings of both linear and volumetric measurements of the maxillary sinus. Maxillary sinus volume was significantly higher in male participants. Maxillary sinus height was the best discriminating parameter for forensic identification with an overall accuracy ranging from 70% to 80%. Conclusions: Maxillary sinus measurements revealed anatomic variability between genders, and this approach can be applied as a complementary method for human identification.

## 1. Introduction

Human identification is a systematic procedure that aims to establish a subject’s personal identity and is important for a variety of reasons such as social, religious and economic [1]. Fingerprints, biological methods such as DNA profiling, and dental evidence are fundamental tools in human identification [2]. The skull and the pelvis are the two osseous structures that most accurately reflect sexual dimorphism [3,4]. Maxillary sinuses in particular, are characterized by their unique feature of maintaining their anatomical shape and structure intact following fire accidents, while other bones may be severely distorted [5]. This specific feature converts maxillary sinus dimensions and volume valuable for forensic purposes [6].

Cone-beam Computed Tomography (CBCT) is a novel digital imaging technology which provides three-dimensional (3D) information about the teeth, including the maxillofacial region, facilitates diagnosis and improves clinical decision-making [7]. Due to the complexity of this anatomical structure, CBCT has emerged as an important adjunctive radiographic method for maxillary sinus evaluation [8].

The aim of this review was to assess scientific evidence published over the last decade to ascertain whether maxillary sinus dimensions and volume may constitute useful parameters for forensic identification and gender determination, based only on cone-beam computed tomography images (CBCT).

## 2. Materials and Methods

### 2.1. Design

The current systematic review of the literature was undertaken to investigate the potential value of the sinuses in gender identification by analyzing data from CBCT.

The Preferred Reporting Items for Systematic Reviews and Meta-Analyses (PRISMA) standards were followed when reporting this review [9]. Computerized literature research with language restrictions was conducted in April 2023 by two of the authors independently (A.M. and N.C.).

### 2.2. Eligibility Criteria

Inclusion and exclusion criteria were applied. 

#### 2.2.1. Types of Participants 

Populations in good health were included. Age, gender, or ethnicity restrictions were not implemented.

#### 2.2.2. Types of Outcome Measures

The study authors created data-collecting forms that listed the most important data from each study (sample size, ethnicity, number of males and females, mean age (range), CBCT planes utilized, measurements performed, software used).

#### 2.2.3. Study Design 

Only human-related research articles and case reports published in English and available in full-text format were considered. In vitro studies and technical comments were also taken into account. Experimental studies that met the criteria for this systematic review were also included.

#### 2.2.4. Inclusion Criteria 

The following inclusion criteria were implemented: full texts of case reports, technical notes, in vitro studies, experimental studies, clinical studies on humans, and articles in the English language published from January 2010 to April 2023. Furthermore, we included only studies that employed CBCT in any field of view. 

#### 2.2.5. Exclusion Criteria

Reviews (systematic and literature), personal views, author arguments, letters to the editor, author replies, books and/or book chapters, newsgroup documents, synopses, editors’ synopses, congress synopses, summary documents, research involving animals, articles published in languages other than English, and papers appraising conventional dentomaxillofacial imaging techniques were all omitted. Papers merging CBCT with other dentomaxillofacial imaging techniques were similarly not included.

### 2.3. Information Sources

The thoroughly searched databases included Scopus, PubMed, Web of Science, and Cochrane Library.

### 2.4. Search Strategy

A determined search was performed to identify any relevant studies based on various combinations of keywords. The aforementioned electronic databases were searched for articles published between January 2010 and April 2023 using the following keywords: “maxillary sinus”, “cone beam computed tomography”, and “forensic identification”. The Boolean operators “AND” and “OR” were used to enhance the search strategy through several combinations. Articles in languages other than English were excluded.

### 2.5. Study Selection 

The process for selecting studies was the following: (A) Duplicate studies retrieved from the aforementioned databases were excluded. (B) Two of the authors (A.M. and N.C.) methodically and independently assessed the titles and identified the papers whose titles fulfilled the study’s objectives. (C) The same reviewers applied the inclusion and exclusion criteria to the abstracts of the selected papers. Articles with titles that fitted the study’s goals but did not have abstracts were completely assessed in the final evaluation. (D) The full texts of the eligible papers were retrieved and reviewed to ensure that they met the eligibility criteria. Before reaching a final conclusion, the authors extensively considered their different views. The causes for rejection were listed separately for each rejected paper. 

### 2.6. Data Collection and Data Items

The data from each study was collected independently by the same two authors (A.M. and N.C.) using a customized and pre-designed data extraction form. The extracted data were compared, and differences were resolved through dialogue and cross-examination of the studies. The following significant study features were included in the data extraction form: sample size, ethnicity, number of males and females, mean age (range), CBCT planes utilized, measurements performed, and software used.

### 2.7. Risk of Bias Assessment in Included Studies

The quality of the included studies was assessed independently by two reviewers (N.C., A.R.) using the ROBINS-I (Risk of Bias in Non-randomized Studies of Interventions) tool for non-randomized trials [10]. In cases of disagreement, a consensus was established after a thorough discussion. The overall RoB level in any of the areas studied determined the overall risk of bias judgment (low, moderate, serious, critical, no information in ROBINS-I) for each study.

In particular, the ROBINS-I tool collects, displays and analyzes accessible data regarding to the risk of bias, in an organized manner in NRSI [10]. For example, the level of expertise of the observers in the evaluation of CBCT images or the sample’s age heterogeneity could lead to a bias due to confounding. Furthermore, using an incorrect evaluation approach may result in a bias in outcome measurement. Consequently, risk of bias assessment is of major importance and a systematic review’s conclusions might be significantly impacted by an insufficient risk of bias assessment. Accordingly, this might lead to recommendations and guidelines that are inaccurate. Thus, when reading systematic reviews, one should be conscious of this problem. Moreover, authors should take care to conduct and disclose risk of bias evaluations in a thorough manner [11].

### 2.8. Effect Measures and Data Synthesis

The main objective of the current systematic review was to observe the importance of maxillary sinus architecture in gender recognition using CBCT images. Author and publication year, gender, mean age, sample size, CBCT planes, software, and outcome assessment instrument (linear/volumetric measures) were the variables identified in each article. 

## 3. Results

### 3.1. Description of Studies

**Figure 1:**
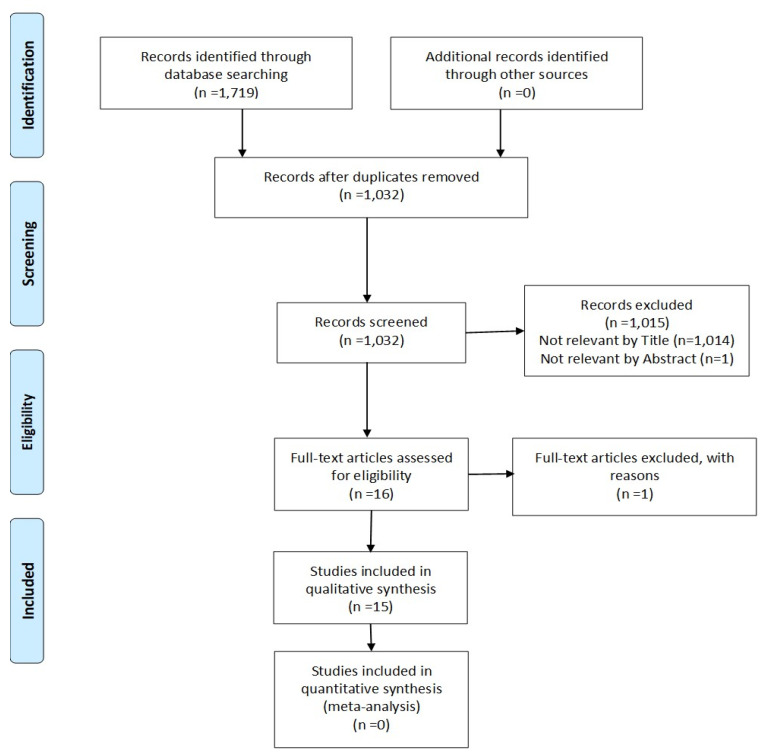
PRISMA flow diagram.

According to the PRISMA statement, a flow diagram should illustrate the outcomes of the literature search and the identification, inclusion, and removal of papers (Figure 1). The initial search revealed 1719 relevant papers, with 1032 remaining after checking for manual duplicates. Following title and abstract screening, fifteen papers were identified for a full analysis based on the inclusion and exclusion criteria [12,13,14,15,16,17,18,19,20,21,22,23,24,25,26].

The articles included were published between 2015 and 2023, with the majority of publications occurring in 2020. Five of the fifteen papers included in the qualitative synthesis reported data from Brazilian populations, four from Turkish populations, two from Indian populations, and one from Italian, Iranian and Indonesian populations. Finally, one paper reported data from Brazilian and Dutch populations [12,13,14,15,16,17,18,19,20,21,22,23,24,25,26]. 

Table 1 summarizes the 15 studies’ overall features and the sample characteristics. There were total of 2475 participants (984 males, 1180 females) in the included studies. One study included 311 subjects; however, it was not specified how many were males and how many were females. The sample populations varied between 52 and 420 individuals. Except for Waluyo et al. (2020), Dhandarany et al. (2023), and Saccucci et al. (2015), who reported results for 84, 80, and 52 participants, respectively, seven studies assessed samples with more than 100 participants, and five of the fifteen articles identified had samples with more than 200 participants. The minimum mean age of the populations studied was 18 ± 6.1 years and the maximum mean age was 50.2 ± 15.6 years. The selected studies used only CBCT images to evaluate morphological and volumetric measurements of the maxillary sinus for human identification [12,13,14,15,16,17,18,19,20,21,22,23,24,25,26]. 

Of the fifteen articles identified, five reported data assessing only volumetric measurements of the maxillary sinus [11,12,13,14,15], seven reported data calculating only linear measurements of maxillary sinus [16,17,18,19,20,21,22], and three reported data by combining findings of both linear and volumetric measurements of the maxillary sinus [24,25,26]. It was impossible to conduct a meta-analysis since every study was evaluated individually and they were heterogeneous.

Wanzeler et al. [15] reported high accuracy rates when evaluating the measurement of the maxillary sinus volume: 96.2% in males and 92.7% in females. Paknahad et al. [17] reported the correct prediction of gender with an accuracy of 74% in males, 78% in females and 76% overall. Mathew and Jacob [18] reported that maxillary sinus height, when used for gender prediction, presented an accuracy of 80%. Dhandapany et al. [25] reported an overall accuracy 84.6% in gender prediction: 89.7% in males and 94.9% in females. The results of this study presented high sensitivity and low specificity. Texeira et al. [20] reported an accuracy in gender prediction of 66.9% when the height of the right maxillary sinus was measured and an accuracy of 64% when the length of the left maxillary sinus was measured. When the measurements of height, width, length, and volume were combined, the overall accuracy of gender prediction was 73.6%. This was the only study in which the specificity and sensitivity of gender determination were evaluated. The highest specificity (52.1%) presented when only the height of the right maxillary sinus was measured. The highest sensitivity (79.8%) presented when the volume of the maxillary sinus was measured.

Of the 15 articles identified, 11 indicated more reliable measurement results for use in forensic medicine [15,17,18,19,20,21,22,23,25,26]. Camba et al. [24] and Ayyildiz and Akgunlu [26], reported that linear maxillary sinus measurements were significantly higher in males. Teixeira et al. [20], Mathew and Jacob [18] and Paknahad et al. [17] reported that the maxillary sinus height was the best discriminant measurement for gender estimation. According to Barros et al. [23], maxillary sinus width was significantly greater in men than in women. However, there were no significant differences in height or depth between the genders. Aşantoğrol and Coşgunarslan [22] and Dhandapany et al. [25] reported that the relationships between gender and the maxillary sinus volume and dimensions were statistically significant. 

Three-dimensional data were registered using diverse types of software programs, e.g., Dolphin Imaging software (Dolphin Imaging and Management solution, Chatsworth, CA, USA) [12], a beta version of the DDS-Pro™ 2.12.0_2021 and 2.14.2_2022 software (DPP Systems, Czestochowa, Poland) [16,21], CS 3D Imaging Software 3.2.9 [18], Xoran software 3.1.62 version (Xoran Technologies, Ann Arbor, MI, USA) [24], iCAT Workstation Dental Imaging System software (XoranCatTM Technology Xoran Technologies) [20], MIMICS 21.0 software (Materialise HQ Technologielaan, Leuven, Belgium) [13], MIMICS 19.0 software [14], ITK-SNAP software (version 2.1.4) [15], Carestream 3D Imaging Software [18], NNT software (NNT, v 3.0; NewTom, Verona, Italy), SimPlant software (v 13.0: Materialize, Leuven, Belgium) [25], OnDemand 3D imaging software (v 3.5.7, Carestream Health Inc., Rochester, NY, USA), and ITK-SNAP software (version 3.8.0) [26], Instrumentarium Dental, Palo DEx Group Oy Nahkelantie 160 FI-04300 TUUSULA, Finland [22].

### 3.2. Risk of Bias

The overall risk of bias for the 15 papers included in this review was rated as moderate using the ROBINS-I tool, as presented in Table 2. All articles were given a moderate risk of bias overall. Six of the included studies appeared to have serious methodological faults due to the lack of a consistent measuring procedure regarding the maxillary sinuses, and the sample size was limited.

## 4. Discussion

Gender determination based on skeletal remains is an important procedure in identification processes [27]. The study of anthropometric characteristics is fundamental to human identification [28]. It has been demonstrated that the maxillary sinuses remain intact in certain cases such as decomposed, burnt, and highly fragmented remains [29] and they can be used as a gender estimation method when conventional gender indicators are absent [30]. Although the dimensions of the maxillary sinus are population-specific, depending on several factors such as race, genetics, maternal health culture, and environmental quality conditions during prenatal development [31], their measurements after puberty have emerged as a reliable factor in gender determination when maxillary sinus reaches its full size [32]. Paknahad et al. [17], Mathew and Jacob [18], Waluyo et al. [21], and Soares et al. [19] reported that measurements of specific morphological and dimensional parameters of maxillary sinus could be valuable for gender determination and thus human identification. The maxillary sinus osseus volume differs depending on gender. Men were shown to have a statistically significant greater mean osseus volume than women [32].

For reliable measurements of maxillary sinus dimensions, CBCT is the perfect imaging modality. Furthermore, CBCT can provide 3D images and accurately assess maxillary sinus volume. As a result, while researching the maxillary sinus area, CBCT should be regarded as the primary approach [33].

In the study by Paknahad et al. [17], three linear measurements of maxillary sinus (height, width, length) were calculated from the coronal and axial planes. Their sample consisted of 100 adult Iranian patients (50 males and 50 females, aged from 20 to 54 years old), who were referred to the Oral and Maxillofacial Radiology Department of Shiraz University of Medical Sciences for various reasons. Individuals with a history of maxillofacial trauma, congenital craniofacial abnormalities, facial asymmetry and individuals with one or more lost maxillary teeth were excluded. Subjects with evidence of any maxillary sinus pathology such as mucosal thickening, sinusitis, or odontogenic cysts, were also excluded after analyzing the CBCT images. According to their statistical analysis (Student’s *t*-test), both height and length showed statistically significant differences between males and females. However, no statistical significance was noted between males and females concerning the width of the maxillary sinuses on both sides. The authors demonstrated higher predictive accuracy in the female population (78%) than in males (74%), with an overall predictive accuracy of 76%. The main limitations of their study were the relatively small sample size and the calculation of measurements by a sole observer.

In the preliminary study of Mathew and Jacob [18], two independent examiners estimated the maxillary sinuses’ dimensions for gender determination. Their sample consisted of 100 adult patients (overall 200 sinuses), selected according to inclusion and exclusion criteria. Independent examiners calculated linear parameters such as width, height, bizygomatic distance, depth, and intermaxillary distance in axial and coronal planes of CBCT images. Although the intraclass correlation coefficient was remarkably high (ranging from 83% to 96%), no statistically significant difference was recorded between the left and right sinus dimensions and intermaxillary distance. The other parameters were statistically significant and significantly greater in males. They also reported that estimating the maxillary sinus’s height in coronal planes was the most accurate parameter and this measurement could be utilized in the field of forensic science. The reliability of the study was limited by the fact that their sample was not divided into subgroups based on age.

In the study by Waluyo et al. [21], statistically significant differences were identified between males and females in measurements of maxillary sinus height, width of the maxillary sinus, and length of maxillary sinus. They also estimated the mandibular canal’s position in relation to adjacent anatomical structures, but no statistically significant differences were observed between males and females. For their study, they selected 84 adult subjects (39 males and 45 females aged between 20 and 65 years) and excluded patients with pathological features, fractures of the maxilla/mandible, and partial edentulousness. Four examiners (three general practitioner dentists under a supervisor who was an experienced radiologist) analyzed 168 CBCT images obtained from DICOM Files (Digital Image Communication in Medicine). Maxillary sinus parameters (width, height, length) were measured using axial, coronal, and axial planes, respectively. Furthermore, they proposed a logistic-regression equation as an additional beneficial tool for gender determination. The application of this equation for gender estimation was limited to individuals younger than 65 years old, while various genetic and environmental factors decreased the maxillary sinus dimensions in the older population.

According to the study by Soares et al. [19], specific parameters such as “total maxillary sinus width” and “distance between the highest points of the sinuses” showed statistically significant differences between genders. For their study, two experienced examiners evaluated 100 CBCT scans (30 males and 70 females, aged between 29 and 87 years old, and between 20 and 79 years old, respectively). Their evaluation included both morphological and dimensional parameter assessments; for the morphological parameters, the examiners performed coronal reconstructions, while for the dimensional parameters, they performed panoramic reconstructions and axial planes. When they disagreed, they consulted axial and sagittal reconstructions. Nevertheless, their method’s validity is limited in several ways. The authors reported divergent results among examiners and, therefore, their solution was to involve a sole forensic examiner in evaluating the CBCT images. Furthermore, the reproducibility of panoramic reconstructions depended on each examiner’s experience.

Wanzeler et al. [15] reported very high rates of success (84,66%) in identifying subjects for forensic purposes. In their study, 163 CBCT scans were analyzed. Individuals who underwent surgery on paranasal sinuses (maxillary, sphenoid and frontal sinuses), and CBCT images that presented pathologies and/or facial deformities were excluded. Two experienced examiners delineated the paranasal sinuses’ surfaces according to their anatomical borders. Volumetric measurements were obtained from axial, coronal, and sagittal CBCT reconstructions using ITK- SNAP software (version 2.1.4). The accuracy rates of gender estimation based on volumetric measurements of the maxillary sinuses were 83.75% (males) and 85.54% (females). The authors proposed a combination of volumetric measurements of paranasal sinuses and foramen magnum measurements, suggesting that this technique can be adopted as a reliable method for the detection of sexual dimorphism in the Brazilian population. The method’s accuracy is limited due to the fact that paranasal sinuses are complex anatomical structures and the delineation of their borders is not always precise.

Teixeira et al. [20] evaluated and measured five different linear parameters (in mm) of the maxillary sinus region (width, length, height, inter-sinus distance, and maximum width). Volume (in mm^3^) of both maxillary sinuses was calculated using the equation V(volume) = (height × width × length) × 0.5, based on Bangi et al.’s study [4]. For their study, they analyzed 420 CBCT scans (adult Brazilian population) and excluded subjects diagnosed with any pathological condition, congenital diseases, craniofacial deformities, history of trauma, fractures or surgery in the maxillary sinus region, sinus pathologies, anatomical bone deformities, and previous orthognathic surgery. According to the study’s results, the best discriminant parameters for gender and age determination were right maximum height (RMH) (66.9%) and inter-sinus distance (ISD) (63.1%). When all parameters were combined, the overall accuracy rates for gender and age estimation were 73.6% and 67.6%, respectively. The authors proposed these specific dimensional and volumetric measurements of maxillary sinus as a complementary method for human identification. A higher accuracy rate was observed in gender assessment, while their method was less effective in age estimation. 

Based on 238 Brazilian patients, including 139 women and 99 men, ranging in age from 6 to 68 years, Barros et al. [23] performed linear measurements in CBCT images. In coronal and sagittal sections, height, width, and depth were measured. Males presented wider maxillary sinuses than women at a statistically significant level. All measurements were higher in males than in females, but in the men, the maxillary width was noticeably broader.

Camba et al. [24] performed CBCT cranial measurements in two different groups, Brazilian and Dutch. Specifically, 311 participants aged 20–60 years were evaluated by CBCT, and seven linear measurements were taken in the maxillary sinuses of both populations. Regardless of population type, the majority of the measurements were greater in males than females at statistically significant levels.

In a retrospective study, Ayyildiz and Akgunlu [26] analyzed CBCT pictures of 212 participants over the age of 18 in order to assess the prevalence of maxillary sinus variation and dimensions, as well as their relationships with age and gender. According to the results of this study, maxillary sinus diameters in males are larger than in females at statistically significant levels.

In contrast, Barros et al. [16], Aktuna Belgin et al. [14], Gulec et al. [13] and Saccucci et al. [12] did not find significant statistical correlations between age or gender and volume measurements of the maxillary sinus. Despite the overall larger maxillary sinus volume in males, this parameter was unreliable for human identification due to the divergence between the actual volume and the volume measured by examiners.

Barros et al. [16] conducted an evaluation of three-dimensional (3D) measurements of the maxillary sinus and aimed to assess a potential correlation between these and specific parameters such as gender, age, skin color, and nutritional status. One-hundred and sixty-one CBCT images of both sexes (72 males and 89 females) were obtained according to the eligibility and exclusion criteria. The Brazilian sample was divided into three age groups (group A: 6–11 years old, group B: 12–17 years old, and group C: older than 18 years). Concerning skin color, individuals were grouped as 120 white subjects and 37 Afro-American subjects. Body Mass Index (BMI) was considered as a variable, with the sample categorized into a subpopulation with normal BMI and a subpopulation with above normal BMI (above 25). DDS-Pro software (version 2.14.2_2022) was used to determine each maxillary sinus‘s region in the sagittal, axial, and coronal planes. Volume and area calculations were performed in a 3D reconstruction based on the final maxillary sinus’s determination. Volumetric measurements of the maxillary sinus (volume and area) did not demonstrate statistically significant differences either between genders or regarding nutritional status. Although these measurements appeared to be higher in white participants and in the male subpopulation, there were no statistically significant differences so they cannot be considered as a reliable indicator of sexual dimorphism.

Gulec et al. [13] investigated the potential correlation between the volumetric measurement of the maxillary sinus and gender estimation using CBCT images in a Turkish subpopulation. DICOM records of 133 patients (49 males, and 84 females) were retrospectively analyzed. CBCT images, obtained from the DICOM format, were transmitted to a personal computer where the volumes of both right and left maxillary sinus were reconstructed and calculated (in cm^3^) using MIMICS 21.0 software. Statistical analysis revealed no statistically significant difference between gender and volume of the maxillary sinus. There was also no statistically significant difference in measurements of the right and left maxillary sinus volumes. The accuracy of measurements with a 3D software program on a DICOM record is less reliable than using an axial, sagittal, and coronal plane as the borders of maxillary sinuses are not uniform, and thus linear measurements are unrealistic.

Dhandapany et al. [25] used volumetric and linear measurements of maxillary sinuses derived from CBCTs together with an Artificial Neural Network (ANN)-based tool to identify the gender of 80 southern Indian individuals. All the CBCT images were subjected to eight linear and two volumetric measurements. The same dataset was uploaded into the ANN software, and the accuracy of gender prediction was assessed. Despite the small sample size, this study provided accurate prediction at rates of up to 89.7% for males and 94.9% for females.

A total of 120 CBCTs from Turkish participants (50 men and 70 women, with a mean age of 22.2 years) were examined by Asantogrol and Cosgunarslan [22]. Specifically, they used the SimPlant software to perform linear and volumetric measurements in axial, sagittal, and coronal sections. The outcomes of this study indicate a statistically significant correlation between gender and the dimensions and volumes of the maxillary sinuses.

Aktuna Belgin et al. [14] conducted a retrospective study to investigate the volumetric changes of the maxillary sinus by age and gender. Two-hundred CBCT images were analyzed. The dataset consisted of axial planes (0.3 mm thickness) as single DICOM records. The region of the maxillary sinus was cropped and delineated according to anatomical borders. The maxillary sinus volume was measured using MIMICS 19.0 (3D software). The sample was divided into five age groups (group A: 18–24 years, (n = 35); group B: 25–34 years, (n = 65); group C: 35–44 years, (n = 50); group D: 45–54 years, (n = 30); and group E: ≥55 years, (n = 20)) and by gender. Both the right and left maxillary sinus volumes of each individual were estimated. The authors found no statistically significant difference between the right and left maxillary sinus volumes and reported that maxillary sinus volume was significantly larger in males than in females. They also mentioned that the maxillary sinus volume varies significantly among age groups. Their results are limited in various ways. There is no reference related to maxillary sinus V measurement correlations at any stage of their retrospective study and their contribution to gender determination and thus to human identification. In addition, they did not mention the exact number of examiners or their level of experience.

Similar results were reported by Saccucci et al. [12]. Their sample consisted of fifty-two individuals whose maxillary sinus volumes were calculated by four independent operators. Volumetric estimation and reproducibility of their technique were performed using Dolphin Imaging software, appropriate for analyzing CBCT scans. According to the study’s results, no statistically significant difference was observed between genders. They proposed that this technique would be more reliable if combined with linear measurements for increasing human identification accuracy.

In the current review, we attempted to research previous literature about three-dimensional measurements (volume and area) of maxillary sinuses obtained from CBCT images and their potential correlation to forensic identification. Many studies have utilized maxillary sinus parameters for identification [4,34,35,36,37]. In our study, the measurement of volume was lower in every female subpopulation than in males [12,13,14,15,16,17,18,19,20,21]. Barros et al. [16], Aktuna Belgin et al. [14], Gulec et al. [13] and Saccucci et al. [12] did not find a significant statistical association between age or gender determination and volume measurement of the maxillary sinus. These results are in agreement with the results of previous studies [38,39,40,41,42,43,44]. The most discriminant measurement for sexual dimorphism was maxillary sinus height with an overall accuracy of 80% [18,21], while Teixeira et al. [20] introduced the “inter-sinuses distance” parameter as an additional individual discriminator that can be applied in gender determination. This result is in agreement with a discrimination analysis performed by Azhar et al. [45]. The study by Texeira et al. [20] was the only one in which the specificity and sensitivity of gender determination were examined. The maximum specificity (52.1%) was seen when only the height of the right maxillary sinus was assessed. When the volume maxillary sinus was measured (either left or right), the sensitivity was at its peak (79.8%). Azhar et al. [45] reported that the left maxillary sinus width was the best discriminating parameter, with an overall accuracy of 61.3%. In contrast, Barros et al. [16], Aktuna Belgin et al. [14], Gulec et al. [13] and Saccucci et al. [12] did not find a significant statistical association between age and maxillary sinus volume. This result is in disagreement with similar studies which were conducted by Vidya et al. [46], Prabhat et al. [47] and Abate et al. [48]. In their studies, the right maxillary sinus volume showed statistically significant differences between males and females, which can be applied in gender estimation [46,47]. Considering the consistent findings of several articles that radiological imaging and (linear) measurements of the maxillary sinus estimated individual’ s gender with an accuracy of up to 70%, it is preferable to combine these results with other forensic evidence due to their limitations. It is logical that gender determination based on anthropometric methods is population-specific and depends on various parameters such as genetics, maternal health culture, environmental conditions, and race [49]. Due to the heterogeneity of the obtained studies, a meta-analysis of the combined information was not possible. Gender estimation plays a vital role in the recomposition of an individual’s biological profile and, ultimately, identification. Given that other significant identification traits, such as age estimation, are linked to gender, assessing gender as accurately as possible is necessary. This highlights the necessity of conducting further high-quality studies with low risk of bias. In particular, equal numbers of both sexes should be represented in samples and bigger samples are required. Furthermore, all data should be documented, and the same parameters should be assessed using comparable statistical tests. Since early studies showed an accuracy in gender estimation of 89.7% for males and up to 94.9% for females, the application of artificial intelligence (AI) to gender determination based on maxillary sinus analysis is a promising approach [25].

## 5. Conclusions

In conclusion, the results of the current review demonstrate that maxillary sinus measurements reveal anatomic variability between genders, and this approach can be applied as a complementary method for human identification. In all the publications reviewed, the volumetric measurements of any female sub-population were smaller than those of their male counterparts. Maxillary sinus height is the dimension that demonstrates the greatest difference between genders. Despite the limited sample size of all studies due to inclusion and exclusion criteria, we recommend combining maxillary sinus measurements with additional forensic evidence when the entire skeleton is unavailable. Further studies are needed to validate these analyses in a broader sample population in order to identify specific features of the morphology of the maxillary sinus.

## Figures and Tables

**Table 1:** Characteristics of included studies in the systematic review.

Authors, Publication Year, Country	Title	Sample Size	Journal	Gender	Mean Age	Planes of CBCT Images	Volume/ Dimension	Software
Saccucci et al., 2015, Italy [12]	Gender assessment through three-dimensional analysis of maxillary sinuses by means of CBCT	52 subjects	Eur Rev Med Pharmacol Sci	27 males, 26 females	24.3 y	3D reconstructions	Volume	Dolphin Imaging software
Paknahad et al., 2017, Iran [17]	Sexual Dimorphism of Maxillary Sinus Dimensions Using Cone-Beam Computed Tomography	100 subjects	J Forensic Sci	50 males, 50 females	34.5 y	Axial, coronal	Dimensional parameters	Not mentioned
Wanzeler et al., 2019, Brazil [15]	Sex estimation using paranasal sinus discriminant analysis: a new approach via cone beam computerized tomography volume analysis	163 subjects	Int J Legal Med	80 males, 83 females	Not mentioned	Axial, sagittal, coronal	Volume	ITK-SNAP (version 2.1.4)
Aktuna Belgin et al., 2019, Turkey [14]	Three-dimensional evaluation of maxillary sinus volume in different age and sex groups using CBCT	200 subjects	Eur Arch Otorhinolaryngol	86 males, 114 females	Not mentioned	Axial	Volume	MIMICS 19.0 (Belgium)
Gulec et al., 2020, Turkey [13]	Three-dimensional volumetric analysis of the maxillary sinus: a cone-beam computed tomography study	133 subjects	Folia Morphol (Warsz)	49 males, 84 females	18 ±6.1 y (females, 19 ± 6.3 y males, 17 ± 5.6 y	3D reconstructions	Volume	MIMICS 21.0 (Belgium)
Teixeira et al., 2020, Brazil [20]	Three-dimensional analysis of the maxillary sinus for determining sex and age in human identification	420 subjects	Forensic Imaging	192 males, 228 females	38.27 ± 15.15 y	Axial, coronal	Volume + linear measurements	Xoran 3.1.62 version (USA)
Soares et al., 2020, Brazil [19]	Morphological and dimensional assessment of the maxillary sinus for human identification and sexual dimorphism: A study using CBCT	100 subjects	Forensic Imaging	30 males, 70 females	51.06 ± 14.32 (males), 46.98 ± 16.48 (females)	Axial, sagittal, coronal, panoramic reconstructions	Dimensional parameters	iCAT Workstation Dental Imaging System
Waluyo et al., 2020, Indonesia [21]	Measurements of sex-related differences in maxillary sinus and mandibular canal characteristic using CBCT	84 subjects	Forensic Imaging	39 males, 45 females	Not mentioned	Axial, coronal	Dimensional parameters	Carestream 3D Imaging
Mathew and Jacob, 2020, India [18]	3D Evaluation of Maxillary Sinus in Gender Determination: A Cone Beam Computed Tomography Study	100 subjects	J Indian Acad Oral Med Radiol	50 males, 50 females	Not mentioned	Axial, coronal	Dimensional parameters	CS 3D Imaging Software 3.2.9
Asantogrol and Cosgunarslan, 2021, Turkey [22]	The effect of anatomical variations of the sinonasal region on maxillary sinus volume and dimensions: a three-dimensional study	120 subjects	Brazilian Journal of Otorhinolaryngology	50 males, 70 females	22.2	Axial, coronal	Volume + linear measurements	NNTsoftware (NNTsoftware, v 3.0; NewTom, Verona, Italy), SimPlant software (v13.0: Materialize, Leuven, Belgium)
Barros et al., 2022a, Brazil [16]	Three-dimensional analysis of the maxillary sinus according to sex, age, skin color, and nutritional status: A study with live Brazilian subjects using cone-beam computed tomography	161 living subjects	Archives of Oral Biology	72 males, 89 females	Not mentioned	Axial, sagittal, coronal	Volume	DDS-Pro (beta version)
Barros et al., 2022b, Brazil [23]	Maxillary sinuses’ height/width/depth of Brazilian subjects and influence of sex, age, skin color, and nutritional status: A CBCT study	238 living subjects	Forensic Imaging	99 males, 139 females	Not mentioned	sagittal, coronal	Linear measurements	DDS-Pro^®^ 2.12.0_2021 software (DPP Systems, Czestochowa, Poland)
Gamba et al., 2023, Brazil and The Netherlands [24]	Comparative study of cranial measurements between sexes from Brazil and The Netherlands: A cone-beam computed tomography study	311 subjects	Journal of Anatomy	Not mentioned	Not mentioned	Axial, coronal	Linear measurements	OnDemand 3D imaging software (CyberMed)
Dhandapany et al., 2023, India [25]	Artificial Neural Network as a Predictive Tool for Gender Determination using Volumetric and Linear Measurements of Maxillary Sinus CBCT: An Observational Study on South Indian Population	80 subjects	Journal of Clinical and Diagnostic Research	40 males, 40 females	Not mentioned	Axial, sagittal, coronal	Volume + linear measurements	CS 3D Imaging software (v 3.5.7, Carestream Health Inc.) and ITK SNAP software (v 3.8.0)
Ayyildiz and Akgunlu, 2023, Turkey [26]	Are maxillary sinus variations related to maxillary sinus diameters?	212 subjects	Oral Radiology	120 males, 92 females	50.2 ± 15.6 y	Axial, sagittal, coronal	Linear measurements	Instrumentarium Dental, Palo DEx Group Oy Nahkelantie 160 FI-04300 TUUSULA, Finland

**Table 2:** Risk of bias of included non-randomized studies according to ROBINS-I tool.

Types of Bias								
Article	Confounding	Selection of Participants for the Study	Classification of Interventions	Deviations from Intended Interventions	Missing Data	Measurement of Outcomes	Selection of the Reported Results	Overall
Saccucci et al., 2015 [12]	Low	Moderate	Low	Low	Low	Low	Low	Moderate
Paknahad et al., 2017 [17]	Moderate	Moderate	Low	Low	Low	Moderate	Moderate	Moderate
Wanzeler et al., 2019 [15]	Moderate	Moderate	Low	Moderate	Low	Moderate	Moderate	Moderate
Aktuna Belgin et al., 2019 [14]	Low	Moderate	Low	Low	Low	Moderate	Moderate	Moderate
Gulec et al., 2020 [13]	Low	Moderate	Low	Moderate	Low	Moderate	Moderate	Moderate
Teixeira et al., 2020 [20]	Low	Moderate	Low	Low	Low	Moderate	Moderate	Moderate
Soares et al., 2020 [19]	Low	Moderate	Low	Moderate	Low	Low	Moderate	Moderate
Waluyo et al., 2020 [21]	Low	Moderate	Low	Low	Low	Low	Moderate	Moderate
Mathew and Jacob 2020 [18]	Low	Moderate	Low	Low	Low	Low	Low	Moderate
Asantogrol and Cosgunarslan 2021 [22]	Low	Moderate	Low	Moderate	Moderate	Low	Low	Moderate
Barros et al., 2022a [16]	Low	Moderate	Low	Moderate	Moderate	Moderate	Moderate	Moderate
Barros et al., 2022b [23]	Low	Moderate	Low	Moderate	Low	Moderate	Moderate	Moderate
Gamba et al., 2023 [24]	Low	Moderate	Low	Moderate	Low	Low	Low	Moderate
Dhandapany et al., 2023 [25]	Low	Moderate	Low	Moderate	Moderate	Moderate	Moderate	Moderate
Ayyildiz and Akgunlu, 2023 [26]	Low	Low	Low	Low	Low	Moderate	Low	Moderate
